# Ethyl 2-(1,2,3,4-tetrahydro­spiro­[carba­zole-3,2′-[1,3]dioxolan]-9-yl)acetate

**DOI:** 10.1107/S160053680900748X

**Published:** 2009-03-06

**Authors:** Philipp M. G. Löffler, Trond Ulven, Andrew D. Bond

**Affiliations:** a University of Southern Denmark, Department of Physics and Chemistry, Campusvej 55, 5230 Odense M, Denmark

## Abstract

In the title compound, C_18_H_21_NO_4_, the hydrogenated six-membered ring of the carbazole unit adopts a half-chair conformation. The dioxolane ring and ethyl­acetate substituent point to opposite sides of the carbazole plane. The ethyl­acetate substituent adopts an essentially fully extended conformation, and its mean plane forms a dihedral angle of 83.8 (1)° with respect to the carbazole mean plane. The mol­ecules are arranged into stacks in which the carbazole planes form a dihedral angle of 4.4 (1)° and have an approximate inter­planar separation of 3.6 Å.

## Related literature

For background literature and synthesis details, see: Ulven & Kostenis (2005 ▶, 2006 ▶). For a related structure, see: Bjerrum *et al.* (2009 ▶).
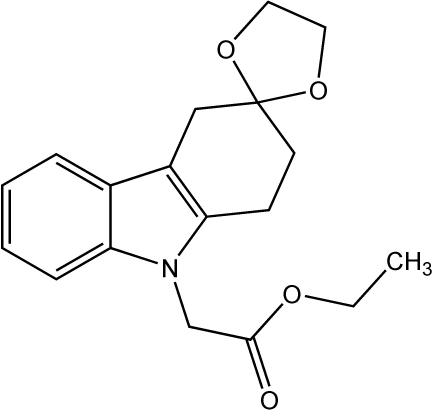


## Experimental

### 

#### Crystal data

C_18_H_21_NO_4_
*M* *_r_* = 315.36
Monoclinic, 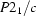

*a* = 10.5533 (4) Å
*b* = 17.3773 (6) Å
*c* = 8.9637 (3) Å
β = 105.629 (1)°
*V* = 1583.05 (10) Å^3^
*Z* = 4
Mo *K*α radiation
μ = 0.09 mm^−1^
*T* = 180 K
0.50 × 0.50 × 0.10 mm

#### Data collection

Bruker-Nonius X8 APEXII CCD diffractometer
Absorption correction: multi-scan (*SADABS*; Sheldrick, 2003 ▶) *T*_min_ = 0.847, *T*_max_ = 0.991
25055 measured reflections
3851 independent reflections
3174 reflections with *I* > 2σ(*I*)
*R*_int_ = 0.025

#### Refinement

*R*[*F* ^2^ > 2σ(*F* ^2^)] = 0.040
*wR*(*F* ^2^) = 0.112
*S* = 1.04
3851 reflections
208 parameters
H-atom parameters constrained
Δρ_max_ = 0.28 e Å^−3^
Δρ_min_ = −0.25 e Å^−3^

### 

Data collection: *APEX2* (Bruker, 2004 ▶); cell refinement: *SAINT* (Bruker, 2003 ▶); data reduction: *SAINT*; program(s) used to solve structure: *SHELXS97* (Sheldrick, 2008 ▶); program(s) used to refine structure: *SHELXL97* (Sheldrick, 2008 ▶); molecular graphics: *SHELXTL* (Sheldrick, 2008 ▶); software used to prepare material for publication: *SHELXTL*.

## Supplementary Material

Crystal structure: contains datablocks global, I. DOI: 10.1107/S160053680900748X/xu2485sup1.cif

Structure factors: contains datablocks I. DOI: 10.1107/S160053680900748X/xu2485Isup2.hkl

Additional supplementary materials:  crystallographic information; 3D view; checkCIF report

## Figures and Tables

**Fig. 1.**
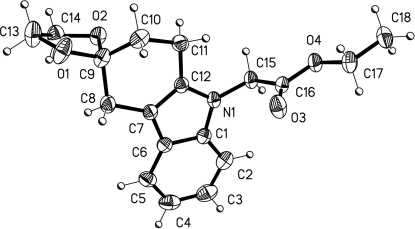
Molecular structure of the title compound with displacement ellipsoids shown at 50% probability for non-H atoms.

**Fig. 2.**
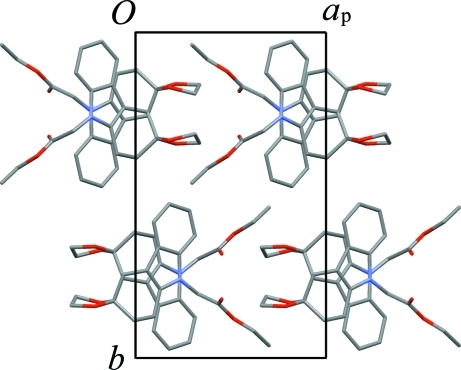
Packing diagram viewed along the c axis, showing stacked carbazole units. H atoms are omitted.

## supplementary crystallographic information

### Comment

The title compound is useful as an intermediate in the synthesis of antagonists of the prostaglandin D_2_ receptor CRTH2 (DP_2_) (Ulven & Kostenis, 2006).

### Experimental

The compound was synthesized as described in Ulven & Kostenis (2005).

### Refinement

H atoms bound to C atoms were placed in idealized positions with C—H = 0.95–0.99 Å and refined as riding with *U*_iso_(H) = 1.2 or 1.5*U*_eq_(C). The methyl group was allowed to rotate about its local threefold axis.

### Crystal data

**Table:**

C_18_H_21_NO_4_	*F*(000) = 672
*M**_r_* = 315.36	*D*_x_ = 1.323 Mg m^−^^3^
Monoclinic, *P*2_1_/*c*	Mo *K*α radiation, λ = 0.71073 Å
Hall symbol: -P 2ybc	Cell parameters from 7203 reflections
*a* = 10.5533 (4) Å	θ = 2.0–28.2°
*b* = 17.3773 (6) Å	µ = 0.09 mm^−^^1^
*c* = 8.9637 (3) Å	*T* = 180 K
β = 105.629 (1)°	Plate, brown
*V* = 1583.05 (10) Å^3^	0.50 × 0.50 × 0.10 mm
*Z* = 4	

### Data collection

**Table:**

Bruker-Nonius X8 APEXII CCD diffractometer	3851 independent reflections
Radiation source: fine-focus sealed tube	3174 reflections with *I* > 2σ(*I*)
graphite	*R*_int_ = 0.025
thin–slice ω and φ scans	θ_max_ = 28.3°, θ_min_ = 3.5°
Absorption correction: multi-scan (*SADABS*; Sheldrick, 2003)	*h* = −13→13
*T*_min_ = 0.847, *T*_max_ = 0.991	*k* = −23→21
25055 measured reflections	*l* = −11→11

### Refinement

**Table:**

Refinement on *F*^2^	Primary atom site location: structure-invariant direct methods
Least-squares matrix: full	Secondary atom site location: difference Fourier map
*R*[*F*^2^ > 2σ(*F*^2^)] = 0.040	Hydrogen site location: inferred from neighbouring sites
*wR*(*F*^2^) = 0.112	H-atom parameters constrained
*S* = 1.04	*w* = 1/[σ^2^(*F*_o_^2^) + (0.0568*P*)^2^ + 0.4296*P*] where *P* = (*F*_o_^2^ + 2*F*_c_^2^)/3
3851 reflections	(Δ/σ)_max_ < 0.001
208 parameters	Δρ_max_ = 0.28 e Å^−^^3^
0 restraints	Δρ_min_ = −0.25 e Å^−^^3^

### Special details

**Table:**

Geometry. All e.s.d.'s (except the e.s.d. in the dihedral angle between two l.s. planes) are estimated using the full covariance matrix. The cell e.s.d.'s are taken into account individually in the estimation of e.s.d.'s in distances, angles and torsion angles; correlations between e.s.d.'s in cell parameters are only used when they are defined by crystal symmetry. An approximate (isotropic) treatment of cell e.s.d.'s is used for estimating e.s.d.'s involving l.s. planes.
Refinement. Refinement of *F*^2^ against ALL reflections. The weighted *R*-factor *wR* and goodness of fit *S* are based on *F*^2^, conventional *R*-factors *R* are based on *F*, with *F* set to zero for negative *F*^2^. The threshold expression of *F*^2^ > σ(*F*^2^) is used only for calculating *R*-factors(gt) *etc*. and is not relevant to the choice of reflections for refinement. *R*-factors based on *F*^2^ are statistically about twice as large as those based on *F*, and *R*- factors based on ALL data will be even larger.

### Fractional atomic coordinates and isotropic or equivalent isotropic displacement parameters (Å^2^)

**Table:**

	*x*	*y*	*z*	*U*_iso_*/*U*_eq_	
O1	1.21234 (9)	0.15818 (7)	1.12008 (10)	0.0460 (3)	
O2	1.17774 (8)	0.16828 (5)	0.85930 (9)	0.0310 (2)	
O3	0.54620 (9)	0.18009 (6)	0.72948 (11)	0.0431 (2)	
O4	0.48419 (8)	0.12028 (5)	0.50032 (10)	0.0319 (2)	
N1	0.76683 (9)	0.24098 (6)	0.66065 (11)	0.0269 (2)	
C1	0.76918 (11)	0.32014 (7)	0.67550 (13)	0.0271 (2)	
C2	0.68683 (13)	0.37599 (8)	0.58858 (16)	0.0375 (3)	
H2A	0.6137	0.3624	0.5048	0.045*	
C3	0.71574 (15)	0.45191 (8)	0.62915 (18)	0.0459 (3)	
H3A	0.6611	0.4912	0.5719	0.055*	
C4	0.82262 (15)	0.47257 (8)	0.75148 (18)	0.0428 (3)	
H4A	0.8399	0.5254	0.7756	0.051*	
C5	0.90393 (13)	0.41716 (7)	0.83833 (15)	0.0339 (3)	
H5A	0.9766	0.4316	0.9219	0.041*	
C6	0.87768 (11)	0.33951 (6)	0.80135 (13)	0.0263 (2)	
C7	0.93961 (10)	0.26886 (6)	0.86264 (13)	0.0256 (2)	
C8	1.06186 (12)	0.25587 (8)	0.99027 (14)	0.0324 (3)	
H8A	1.0427	0.2630	1.0916	0.039*	
H8B	1.1296	0.2940	0.9825	0.039*	
C9	1.11382 (11)	0.17469 (7)	0.98003 (13)	0.0305 (3)	
C10	1.00563 (12)	0.11489 (7)	0.95185 (14)	0.0334 (3)	
H10A	0.9625	0.1170	1.0371	0.040*	
H10B	1.0448	0.0631	0.9526	0.040*	
C11	0.90183 (12)	0.12724 (7)	0.79701 (14)	0.0311 (3)	
H11A	0.9359	0.1086	0.7109	0.037*	
H11B	0.8213	0.0977	0.7958	0.037*	
C12	0.86989 (10)	0.21090 (6)	0.77637 (13)	0.0255 (2)	
C13	1.33628 (14)	0.16069 (11)	1.09153 (17)	0.0486 (4)	
H13A	1.3774	0.1090	1.1043	0.058*	
H13B	1.3950	0.1968	1.1636	0.058*	
C14	1.31189 (12)	0.18790 (9)	0.92705 (15)	0.0398 (3)	
H14A	1.3257	0.2441	0.9228	0.048*	
H14B	1.3704	0.1612	0.8740	0.048*	
C15	0.67326 (11)	0.19717 (7)	0.54503 (13)	0.0290 (2)	
H15A	0.7192	0.1537	0.5107	0.035*	
H15B	0.6361	0.2304	0.4540	0.035*	
C16	0.56219 (11)	0.16596 (6)	0.60498 (13)	0.0264 (2)	
C17	0.37837 (12)	0.08256 (8)	0.54942 (15)	0.0373 (3)	
H17A	0.3230	0.1215	0.5826	0.045*	
H17B	0.4156	0.0477	0.6377	0.045*	
C18	0.29786 (13)	0.03789 (8)	0.41509 (16)	0.0407 (3)	
H18A	0.2259	0.0120	0.4450	0.061*	
H18B	0.3534	−0.0006	0.3835	0.061*	
H18C	0.2613	0.0729	0.3285	0.061*	

### Atomic displacement parameters (Å^2^)

**Table:**

	*U*^11^	*U*^22^	*U*^33^	*U*^12^	*U*^13^	*U*^23^
O1	0.0307 (5)	0.0794 (7)	0.0284 (5)	0.0149 (5)	0.0090 (4)	0.0144 (4)
O2	0.0270 (4)	0.0428 (5)	0.0261 (4)	0.0009 (3)	0.0119 (3)	0.0002 (3)
O3	0.0339 (5)	0.0658 (6)	0.0338 (5)	−0.0159 (4)	0.0163 (4)	−0.0132 (4)
O4	0.0255 (4)	0.0388 (5)	0.0317 (4)	−0.0088 (3)	0.0081 (3)	−0.0039 (3)
N1	0.0220 (5)	0.0306 (5)	0.0275 (5)	−0.0043 (4)	0.0057 (4)	−0.0010 (4)
C1	0.0241 (5)	0.0308 (6)	0.0295 (5)	−0.0023 (4)	0.0126 (4)	0.0011 (4)
C2	0.0323 (6)	0.0439 (7)	0.0371 (6)	0.0055 (5)	0.0106 (5)	0.0077 (5)
C3	0.0507 (8)	0.0371 (7)	0.0540 (8)	0.0124 (6)	0.0211 (7)	0.0119 (6)
C4	0.0532 (8)	0.0284 (6)	0.0553 (8)	0.0005 (6)	0.0294 (7)	−0.0007 (6)
C5	0.0356 (6)	0.0329 (6)	0.0385 (6)	−0.0058 (5)	0.0193 (5)	−0.0072 (5)
C6	0.0247 (5)	0.0302 (6)	0.0280 (5)	−0.0022 (4)	0.0138 (4)	−0.0017 (4)
C7	0.0227 (5)	0.0307 (6)	0.0256 (5)	−0.0016 (4)	0.0100 (4)	−0.0027 (4)
C8	0.0264 (6)	0.0425 (7)	0.0270 (6)	0.0004 (5)	0.0051 (4)	−0.0068 (5)
C9	0.0264 (6)	0.0444 (7)	0.0221 (5)	0.0054 (5)	0.0092 (4)	0.0046 (5)
C10	0.0331 (6)	0.0362 (6)	0.0350 (6)	0.0046 (5)	0.0160 (5)	0.0083 (5)
C11	0.0299 (6)	0.0284 (6)	0.0361 (6)	−0.0026 (4)	0.0109 (5)	−0.0010 (5)
C12	0.0216 (5)	0.0300 (6)	0.0263 (5)	−0.0019 (4)	0.0088 (4)	−0.0005 (4)
C13	0.0301 (7)	0.0766 (11)	0.0369 (7)	−0.0055 (7)	0.0054 (5)	−0.0011 (7)
C14	0.0288 (6)	0.0552 (8)	0.0383 (7)	−0.0054 (6)	0.0142 (5)	−0.0041 (6)
C15	0.0241 (5)	0.0381 (6)	0.0252 (5)	−0.0066 (5)	0.0073 (4)	−0.0031 (5)
C16	0.0204 (5)	0.0315 (6)	0.0265 (5)	−0.0005 (4)	0.0050 (4)	0.0002 (4)
C17	0.0277 (6)	0.0442 (7)	0.0404 (7)	−0.0096 (5)	0.0099 (5)	0.0018 (6)
C18	0.0340 (7)	0.0403 (7)	0.0445 (7)	−0.0114 (5)	0.0049 (5)	0.0023 (6)

### Geometric parameters (Å, °)

**Table:**

O1—C13	1.3995 (17)	C8—H8A	0.990
O1—C9	1.4268 (14)	C8—H8B	0.990
O2—C14	1.4233 (15)	C9—C10	1.5140 (18)
O2—C9	1.4254 (13)	C10—C11	1.5340 (17)
O3—C16	1.1982 (14)	C10—H10A	0.990
O4—C16	1.3311 (14)	C10—H10B	0.990
O4—C17	1.4613 (14)	C11—C12	1.4926 (16)
N1—C1	1.3817 (15)	C11—H11A	0.990
N1—C12	1.3872 (14)	C11—H11B	0.990
N1—C15	1.4412 (14)	C13—C14	1.503 (2)
C1—C2	1.3926 (17)	C13—H13A	0.990
C1—C6	1.4146 (16)	C13—H13B	0.990
C2—C3	1.381 (2)	C14—H14A	0.990
C2—H2A	0.950	C14—H14B	0.990
C3—C4	1.392 (2)	C15—C16	1.5154 (15)
C3—H3A	0.950	C15—H15A	0.990
C4—C5	1.382 (2)	C15—H15B	0.990
C4—H4A	0.950	C17—C18	1.4910 (18)
C5—C6	1.3993 (16)	C17—H17A	0.990
C5—H5A	0.950	C17—H17B	0.990
C6—C7	1.4293 (16)	C18—H18A	0.980
C7—C12	1.3591 (16)	C18—H18B	0.980
C7—C8	1.4929 (15)	C18—H18C	0.980
C8—C9	1.5252 (18)		
			
C13—O1—C9	109.16 (9)	H10A—C10—H10B	107.9
C14—O2—C9	106.09 (9)	C12—C11—C10	109.32 (10)
C16—O4—C17	115.65 (9)	C12—C11—H11A	109.8
C1—N1—C12	108.24 (9)	C10—C11—H11A	109.8
C1—N1—C15	125.89 (10)	C12—C11—H11B	109.8
C12—N1—C15	125.87 (10)	C10—C11—H11B	109.8
N1—C1—C2	130.37 (11)	H11A—C11—H11B	108.3
N1—C1—C6	107.66 (10)	C7—C12—N1	109.97 (10)
C2—C1—C6	121.97 (11)	C7—C12—C11	125.57 (10)
C3—C2—C1	117.28 (13)	N1—C12—C11	124.41 (10)
C3—C2—H2A	121.4	O1—C13—C14	105.53 (11)
C1—C2—H2A	121.4	O1—C13—H13A	110.6
C2—C3—C4	121.92 (13)	C14—C13—H13A	110.6
C2—C3—H3A	119.0	O1—C13—H13B	110.6
C4—C3—H3A	119.0	C14—C13—H13B	110.6
C5—C4—C3	120.83 (13)	H13A—C13—H13B	108.8
C5—C4—H4A	119.6	O2—C14—C13	103.35 (10)
C3—C4—H4A	119.6	O2—C14—H14A	111.1
C4—C5—C6	119.03 (12)	C13—C14—H14A	111.1
C4—C5—H5A	120.5	O2—C14—H14B	111.1
C6—C5—H5A	120.5	C13—C14—H14B	111.1
C5—C6—C1	118.98 (11)	H14A—C14—H14B	109.1
C5—C6—C7	134.14 (11)	N1—C15—C16	112.31 (9)
C1—C6—C7	106.87 (10)	N1—C15—H15A	109.1
C12—C7—C6	107.24 (10)	C16—C15—H15A	109.1
C12—C7—C8	123.20 (11)	N1—C15—H15B	109.1
C6—C7—C8	129.47 (10)	C16—C15—H15B	109.1
C7—C8—C9	110.13 (10)	H15A—C15—H15B	107.9
C7—C8—H8A	109.6	O3—C16—O4	124.27 (10)
C9—C8—H8A	109.6	O3—C16—C15	124.99 (10)
C7—C8—H8B	109.6	O4—C16—C15	110.74 (9)
C9—C8—H8B	109.6	O4—C17—C18	107.80 (10)
H8A—C8—H8B	108.1	O4—C17—H17A	110.1
O2—C9—O1	105.70 (9)	C18—C17—H17A	110.1
O2—C9—C10	108.03 (10)	O4—C17—H17B	110.1
O1—C9—C10	110.29 (10)	C18—C17—H17B	110.1
O2—C9—C8	111.67 (10)	H17A—C17—H17B	108.5
O1—C9—C8	108.76 (10)	C17—C18—H18A	109.5
C10—C9—C8	112.19 (10)	C17—C18—H18B	109.5
C9—C10—C11	112.18 (10)	H18A—C18—H18B	109.5
C9—C10—H10A	109.2	C17—C18—H18C	109.5
C11—C10—H10A	109.2	H18A—C18—H18C	109.5
C9—C10—H10B	109.2	H18B—C18—H18C	109.5
C11—C10—H10B	109.2		
			
C12—N1—C1—C2	−179.74 (11)	C7—C8—C9—O2	76.13 (12)
C15—N1—C1—C2	0.15 (19)	C7—C8—C9—O1	−167.60 (9)
C12—N1—C1—C6	1.15 (12)	C7—C8—C9—C10	−45.32 (13)
C15—N1—C1—C6	−178.96 (9)	O2—C9—C10—C11	−61.20 (12)
N1—C1—C2—C3	−178.46 (12)	O1—C9—C10—C11	−176.27 (9)
C6—C1—C2—C3	0.54 (18)	C8—C9—C10—C11	62.32 (13)
C1—C2—C3—C4	0.1 (2)	C9—C10—C11—C12	−43.12 (13)
C2—C3—C4—C5	−0.5 (2)	C6—C7—C12—N1	0.87 (12)
C3—C4—C5—C6	0.20 (19)	C8—C7—C12—N1	−176.05 (9)
C4—C5—C6—C1	0.41 (16)	C6—C7—C12—C11	178.28 (10)
C4—C5—C6—C7	179.11 (12)	C8—C7—C12—C11	1.36 (17)
N1—C1—C6—C5	178.40 (10)	C1—N1—C12—C7	−1.28 (12)
C2—C1—C6—C5	−0.80 (16)	C15—N1—C12—C7	178.83 (10)
N1—C1—C6—C7	−0.62 (11)	C1—N1—C12—C11	−178.72 (10)
C2—C1—C6—C7	−179.82 (10)	C15—N1—C12—C11	1.39 (16)
C5—C6—C7—C12	−178.95 (12)	C10—C11—C12—C7	12.73 (15)
C1—C6—C7—C12	−0.15 (12)	C10—C11—C12—N1	−170.22 (10)
C5—C6—C7—C8	−2.3 (2)	C9—O1—C13—C14	5.60 (16)
C1—C6—C7—C8	176.51 (11)	C9—O2—C14—C13	31.96 (14)
C12—C7—C8—C9	14.73 (15)	O1—C13—C14—O2	−23.07 (16)
C6—C7—C8—C9	−161.45 (11)	C1—N1—C15—C16	−97.30 (13)
C14—O2—C9—O1	−29.21 (13)	C12—N1—C15—C16	82.58 (13)
C14—O2—C9—C10	−147.26 (10)	C17—O4—C16—O3	−3.87 (17)
C14—O2—C9—C8	88.91 (12)	C17—O4—C16—C15	176.02 (10)
C13—O1—C9—O2	14.11 (14)	N1—C15—C16—O3	5.41 (17)
C13—O1—C9—C10	130.64 (12)	N1—C15—C16—O4	−174.47 (9)
C13—O1—C9—C8	−105.93 (13)	C16—O4—C17—C18	176.84 (10)

## References

[bb1] Bjerrum, J. V., Ulven, T. & Bond, A. D. (2009). *Acta Cryst.* E**65**, o579. 10.1107/S1600536809005558 PMC2968561 21582234

[bb2] Bruker (2003). *SAINT* Bruker AXS Inc., Madison, Wisconsin, USA.

[bb3] Bruker (2004). *APEX2* Bruker AXS Inc., Madison, Wisconsin, USA.

[bb4] Sheldrick, G. M. (2003). *SADABS* Bruker AXS Inc., Madison, Wisconsin, USA.

[bb5] Sheldrick, G. M. (2008). *Acta Cryst.* A**64**, 112–122. 10.1107/S0108767307043930 18156677

[bb6] Ulven, T. & Kostenis, E. (2005). *J. Med. Chem.* **48**, 897–900. 10.1021/jm049036i 15715457

[bb7] Ulven, T. & Kostenis, E. (2006). *Curr. Top. Med. Chem.* **6**, 1427–1444. 10.2174/15680266106061427 16918458

